# Immune modulation by sex hormones: unveiling the complex interplay and regulatory networks

**DOI:** 10.3389/fimmu.2026.1748097

**Published:** 2026-02-26

**Authors:** Bohan Wan, Wanquan Chen, Mengmeng Shan, Liang Chi

**Affiliations:** 1 Department of Endocrinology, Nanjing Drum Tower Hospital, Affiliated Hospital of Medical School, National Resource Center for Mutant Mice, Jiangsu Key Laboratory of Molecular Medicine, Model Animal Research Center, Medical School of Nanjing University, Nanjing, China; 2 Department of Pediatrics, The First Affiliated Hospital of Harbin Medical University, Harbin, China; 3 Ruijin-Hainan Hospital, Shanghai Jiao Tong University School of Medicine (Hainan Boao Research Hospital), Qionghai, China

**Keywords:** sex differences, sex hormones, immune response, pregnancy, aging, hormone disruptors

## Abstract

Sex hormones, androgens, estrogens, and progestogens, play critical regulatory roles in the development and activation of the immune system, which contribute to the sex dimorphism in the disease susceptibility. Sex hormone receptors are expressed in various of immune cells as well as epithelial and stromal cells, implying both direct and indirect effects of sex hormones. Immune system homeostasis can be disrupted by fluctuations in sex hormone levels, which occur during physiological states such as pregnancy and aging, under the influence of the microbiota, or upon exposure to endocrine disruptors. All of these factors have potential impacts on the immune system homeostasis. Here, we provide an overview of the sex hormone system and how sex hormones affect the immune system. Additionally, we highlight the roles of epithelial and stromal cells in the sex hormone-immune crosstalk and discuss key factors that affect sex hormone levels and have potential regulatory effects on immune balance.

## 1 Introduction

Sexual dimorphism in disease susceptibility has been extensively observed in the contexts of various of diseases. Generally, females are more sensitive to autoimmune diseases, such as Systemic lupus erythematosus (SLE), with a ratio of 6:1 in incidence and a ratio of 10:1 in prevalence, while males are more susceptible to cancers and infectious diseases. For example, during the past COVID-19 pandemic, male-biased mortality was commonly observed in studies worldwide (1). Sex hormones play an essential role in reproduction and sexual development, but they are also key mediators that drive sex-biased disease susceptibilities and outcomes. For instance, the incidence of SLE correlates with estrogen levels, with both premenopausal and menopausal women experiencing low incidences of SLE (2). However, pregnancy, which involves a dramatic increase in estrogen levels, can exacerbate the severity of SLE (3). Estrogen also promotes airway inflammation and can cause severe asthma, while androgens inhibit airway inflammation in asthma (4).

The immune system protects us from various external challenges and significantly influences disease outcomes. The effects of sex hormones on the immune system have been explored since the last century, with numerous studies revealing that sex hormones regulate the activity and function of immune cells (5). For example, an early study found that estrogen affects CD4^+^ T cell activity resulting in sex-related disparity in susceptibility to meningitis (6). Sex hormones regulate cellular gene expression primarily by activating sex hormone receptors which function as ligand-dependent transcriptional factors (7). Upon hormone binding, these receptors undergo conformational changes, dimerize, and translocate to the nucleus, where they bind to specific hormone response elements (HREs) in DNA to modulate target gene transcription (7, 8). Accumulating evidence indicates that sex hormone receptors are expressed in various of immune cells, allowing hormones to directly regulate immune cells. For instance, androgen receptor is expressed in type 2 innate lymphoid cells (ILC2) (9). Androgens negatively regulate ILC2 activity in the lung reducing the severity of airway inflammation (10, 11).

A comprehensive understanding of sex hormones as immune regulators is therefore crucial, not only to elucidate sex-based differences in diseases but also to advance our current knowledge of immune cell biology across sexes. In this review, we summarize established findings and underlying mechanisms of sex hormone–immune interactions. We further explore the underappreciated role of stromal cells in mediating tissue-specific crosstalk, and examine how internal and external factors that perturb hormone homeostasis can reshape immune responses. Insights gained from these discussions will enhance our understanding of sex hormone functions in immune homeostasis, providing a foundation for further research to develop more tailored therapeutic strategies.

## 2 Sex hormone biosynthesis, dynamics, and working model

### 2.1 Synthesis of sex hormones

**Figure 1:**
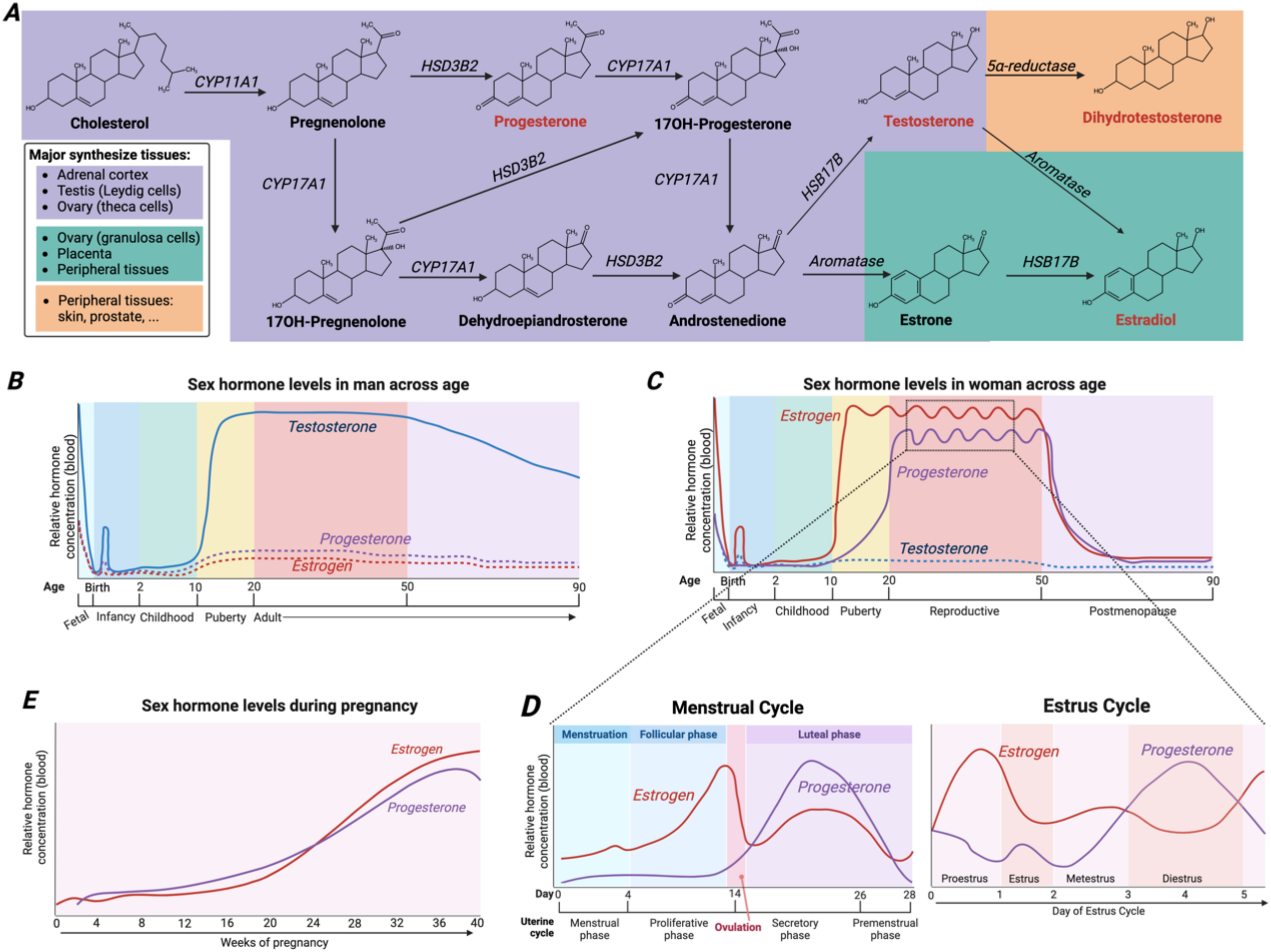
Sex hormone synthesis and fluctuation within the human body. **(A)** Classical pathways and enzymes of *de novo* sex hormone synthesis. **(B–E)** Sex hormone levels in males **(B)** and females **(C)** at different ages. **(D)** Levels of estrogen and progesterone in the blood of females throughout the menstrual cycle and estrus cycle. **(E)** Levels of estrogen and progesterone in the blood during pregnancy.

Sex hormones, including androgens, estrogens, and progestogens, are crucial for sexual development, reproduction, and broad physiological processes including immune function (12). Their synthesis is primarily governed by the hypothalamic–pituitary–gonadal (HPG) axis within the gonads, where cholesterol is converted via a conserved enzymatic cascade into key intermediates and final hormones (**Figure 1A**). In response to signals from luteinizing hormone, Leydig cells in the testis and theca cells in the ovary utilize cholesterol to synthesize sex hormones. Briefly, cholesterol is transported into mitochondria and cleaved to form pregnenolone, which is then metabolized into progesterone, dehydroepiandrosterone (DHEA), and androstenedione by enzymes including 3β-hydroxysteroid dehydrogenase (3β-HSD) and 17α-hydroxylase. Androstenedione serves as the direct precursor for testosterone in males and estradiol in females (**Figure 1A**).

In addition to gonadal synthesis, the adrenal glands produce significant amounts of hormone precursors such as DHEA and androstenedione, using the same biochemical pathway. These precursors enter the circulation and can be converted into active hormones in peripheral tissues. Importantly, many peripheral sites, such as skin, breast, brain, and blood vessels, express enzymes such as aromatase, 17β-hydroxysteroid dehydrogenase (17β-HSD), and 5α-reductase, enabling local synthesis of potent hormones like dihydrotestosterone (DHT) and estradiol. For example, testosterone can be aromatized to estradiol in several tissues under follicle-stimulating hormone (FSH) stimulation. During pregnancy, the placenta becomes a major endocrine organ, producing large quantities of estrogens and progesterone that are essential for maintaining pregnancy and supporting fetal development (13).

### 2.2 Levels of sex hormones in the human body

Sex hormone levels undergo dynamic changes throughout life (**Figures 1B, C**). An early fetal surge, peaking around week 6 of gestation, promotes gonadal development before declining by week 20 (14). After birth, infants go through a stage known as minipuberty (15, 16). In boys, testosterone reaches its peak during the 2nd to 3rd month of life, and in girls, estradiol levels are elevated in the first month. These early elevations are critical for the development of genital organs and can also influence the development of other organs, such as the brain (16). Following minipuberty, sex hormones return to prepubertal levels by around 6 months and remain relatively low during childhood until the onset of puberty. During puberty, sex hormone levels steadily increase. In females, estrogens and progesterone increase, while in males, androgens (along with a low level of progesterone) increase. Post-maturation, males exhibit a gradual age-related decline in androgens (**Figure 1B**), whereas females experience marked cyclical and life-stage fluctuations (**Figures 1C–E**). Firstly, during the menstrual cycle (approximately 28-day), estrogen rises in the mid-follicular phase, drops after ovulation, rises again at mid-luteal phase, and then falls. Progesterone levels increase after ovulation and decline at the pre-menstrual phase. Pregnancy drives a continuous rise in estrogens and progestogens which are largely contribute by the placenta and ovary (**Figure 1E**), followed by a sharp postpartum drop. In contrast to gradual declining of androgens throughout males’ aging, menopause in females brings a dramatic, sustained decline in estrogens and progesterone (**Figures 1C, D**).

Importantly, circulating hormone levels do not equal bioactive levels; only free hormones activate receptors. In mammals, most of the sex hormones in circulation bind to proteins, primarily serum albumin and sex hormone-binding globulin (SHBG). Albumin, produced by the liver, is the most abundant protein in human blood. Albumin binds to sex hormone molecules non-selectively and with relatively low affinity. SHBG, a glycoprotein also produced by the liver and secreted into the bloodstream, binds to estrogens and androgens with high affinity. These proteins extend hormone half-life but limit tissue bioavailability (17). SHBG levels are key determinants of free hormone activity. Hepatic SHBG production begins in fetal life, aligns with hormonal changes during sexual differentiation and remains high until puberty (17), after which plasma SHBG levels significantly decline to supporting sexual maturation (18, 19). Post-puberty, SHBG levels increase but are individually variable, influenced by genetics, nutrition, and metabolic state (20). During pregnancy, maternal SHBG rises levels 5-10-fold, which might protect against fetal androgens as lower SHBG production correlates with hyperandrogenism and ovarian dysfunction (21). SHBG also fluctuates with estradiol across the menstrual cycle (22). Therefore, both hormone and SHBG levels vary substantially across developmental and physiological stages, which could further affect immune homeostasis.

### 2.3 Sex hormone receptors and their mechanisms of action

**Figure 2:**
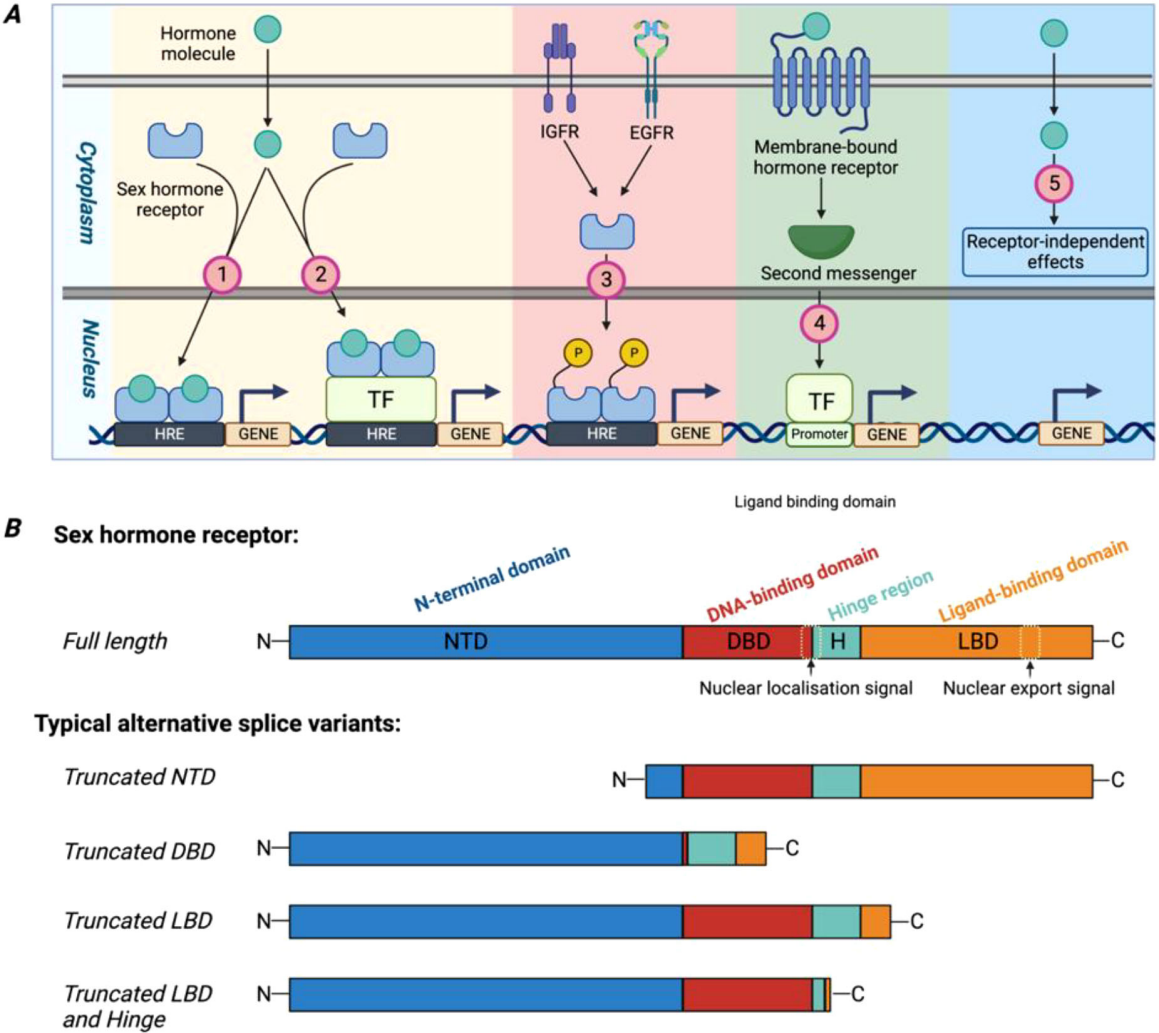
Working mechanisms of sex hormone receptors regulating gene expression and cell activities. **(A)** Five different approaches through which sex hormone receptors coordinate gene expression in cells. **(B)** Structural composition of typical sex hormone receptors and several alternative splice variants.

The classical genomic pathway of sex hormone action involves ligand binding to specific nuclear receptors—estrogen receptors (ERα/ERβ), androgen receptor (AR), progesterone receptor (PGR; progesterone can also activate the glucocorticoid receptor, GR)—which then function as transcription factors (**Figure 2A**). Upon activation, receptors translocate to the nucleus and bind to specific hormone response elements to regulate target-gene transcription (**Figure 2A**). Typical HRE motifs include ERE (estrogen response element), ARE (androgen response element), and PRE (progesterone response element), with sequences like 5′-GGTCAnnnTGACC-3′ (23), 5′-GGTAC A/G CGGTGTTCT-5′, and 5′-G/A G G/T AC A/G TGGTGTTCT-3′ (24), respectively. They can also cooperate with other transcription factors (25, 26), such as AP-1 proteins, NF-κB, Sp-1, and p53, or be phosphorylated and activated via cross-talk with growth-factor receptors such as EGFR and IGF1R, enabling both estrogen-dependent and estrogen-independent transcriptional regulation (27, 28).

Sex hormone receptors also mediate transcription-independent non-genomic actions (**Figure 2A**). These are initiated through membrane-associated receptors or via cytosolic kinase cascades, influencing processes such as proliferation, apoptosis and migration (29). For instance, in MCF-7 breast cancer cells, estradiol binding to ERα, triggered the Src/PI3-K/Akt pathway to promote DNA synthesis (30, 31). Likewise, androgen-AR signaling activates Erk-2 to drive proliferation (32). Non-genomic actions of sex hormones are often initiated by binding to membrane-associated receptors, notably G protein-coupled receptors such as the estradiol receptor GPR30 (33). Ligand binding activates downstream second messenger pathways, such as cAMP/PKA signaling and PI3K/AKT signaling (34–36).

The distinct biological activities of different receptor isomers and splice variants of sex hormone receptors introduce another layer of complexity to the effects of sex hormones (**Figure 2B**). It has been reported that ERα and ERβ differ in tissue distribution, ligand affinity, and sometimes transcriptional outcomes. ERα predominantly expressed in reproductive tissues (uterus and ovary), breast, kidney, adipose tissue, and liver, while ERβ is highly expressed in tissues such as the ovary, central nervous system, cardiovascular system, lung, colon, kidney, and the immune system. ERα and ERβ possess different amino acid sequences in ligand binding domains, resulting in differing affinities for ligands. Estrogen elicits opposing transcriptional outcomes at an AP-1 site, depending on the receptor subtype, activating transcription through ERα but repressing it through ERβ (37).

Alternative splicing generates numerous receptor variants (**Figure 2B**). For instance, via exon duplication (38), insertion (39) or deletion, both ERα and ERβ have numerous splice variants such as ERα-S, ERβ-M, ERαΔ5, and ERαΔ7. A similar phenomenon is observed in PGR (40) and the AR (41). Most splice variants lack intact ligand- or DNA-binding domains but can modulate wild-type receptor activity (42). A representative example is the ERαΔ3 variant, which binds estrogen but fails to activate transcription due to a truncated DNA-binding domain, thereby inhibiting full-length ERα (43). The AR-V7 splice variant, lacking the ligand-binding domain but retaining DNA-binding and nuclear-import signals, is constitutively active and upregulates cell-cycle genes, often found in prostate tumors (44). Furthermore, even similarly truncated variants may display different capacities to regulate AR-target genes, such as mAR-V2 and mAR-V4 (45).

A limited number of receptor-independent effects have also been reported (**Figure 2A**). For instance, estradiol and its metabolites inhibit endothelin-1 synthesis and neointima formation, protecting against cardiovascular diseases independently of ER (46). Low-concentration estradiol may induce neoplastic transformation of ER-negative MCF-10F cells (47). Androgens can influence prostate cancer and atherosclerosis in AR-independent manners (48, 49). The mechanisms underlying these pathways, however, remain poorly defined and require further investigation.

## 3 Sex hormone effects on the immune system

### 3.1 Sex hormone effects on different immune cells

**Figure 3:**
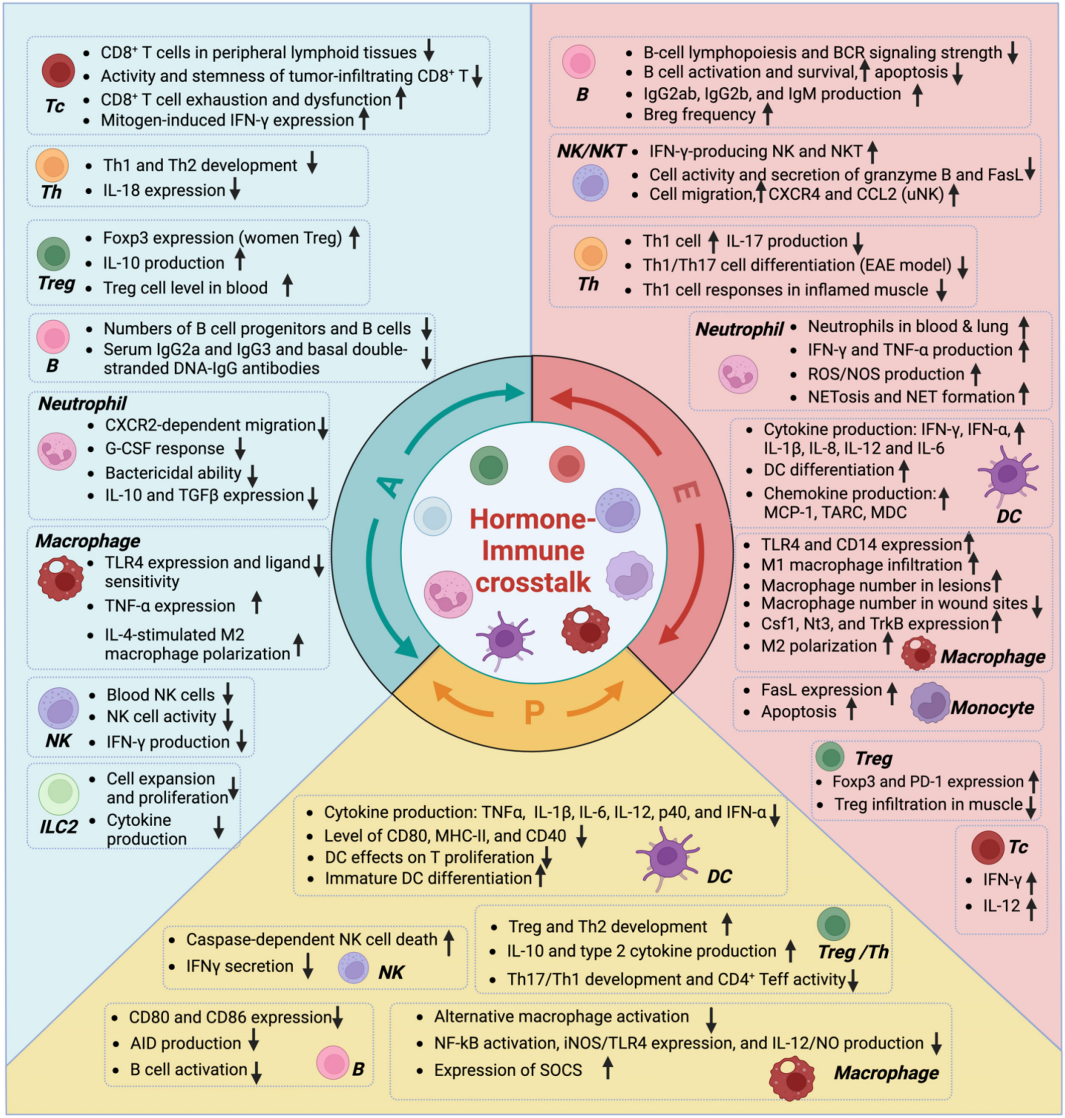
Effects of androgens, estrogens, and progestogens in different immune cells.

The sexual dimorphism of the immune system and the pivotal role of sex hormones have been documented since last century (50, 51). Broadly speaking, estrogens and androgens have been broadly characterized as exerting different immunomodulatory effects, with estrogens enhancing immune activation and androgens suppressing it. However, extensive research over recent decades has revealed a far more complex landscape. The influence of each major sex hormone—estrogens, androgens, and progestogens—on the development, polarization, and effector functions of immune cells is profound, yet highly context-dependent, varying with cell type, tissue microenvironment, receptor expression, and hormonal concentration. Here, we summarize key, representative findings to provide an integrated overview of these interactions (**Figure 3**). The specific sex hormone effects on immune system are also well-summarized in several dedicated reviews (5, 52–54).

#### 3.1.1 Androgens

The effects of androgens on the immune system have been investigated in many previous studies and are known for their anti-inflammatory effects. First, androgens play a role in regulating thymus and T cell development. The androgen receptor (AR) is detected in hematopoietic progenitors, and androgen levels influence thymus size, thymocyte proliferation, and apoptosis (55–59). Androgen deprivation or castration leads to thymus enlargement, while testosterone administration causes thymus atrophy (55–61). Androgens also upregulate the expression of the autoimmune regulator (Aire) in medullary thymic epithelial cells (mTECs), enhancing the negative selection process (62). Castration increases the emigration of T cells from the thymus to the periphery, expanding the pool of peripheral T cell populations. Correspondingly, *in vitro* treatment with DHT can induce cell death (55, 57, 63). Androgens have differential effects on T cell populations. Administration of androgens to females increases the level of Treg by upregulating *Foxp3* expression (64) and promotes the production of anti-inflammatory cytokines such as IL-10 and TGF-β (65). Androgen signaling also inhibits Th1 differentiation by decreasing IL-12-induced STAT4 phosphorylation (66). Recent studies have focused on the effects of androgens on CD8^+^ T cells, particularly in the context of cancer. It has been found that AR signaling promotes CD8^+^ T cell exhaustion and reduces anti-tumor immunity (67, 68). In contrast, depleting AR signaling enhances the function of CD8^+^ T cells by increasing IFN-γ production and responsiveness to PD-1 inhibiting therapy. A recent study identified an endogenous bile acid AR antagonist which could boost anti-tumor immunity by inhibiting AR signaling in CD8^+^ T cells (69).

The inhibitory effects of androgens on innate immune cells have also been studied. For example, androgens negatively regulate macrophage activation, including the decrease in TLR4 and iNOS expression, as well as TNF-α, IL-1β, and NO production (70–72). In addition, androgens promote the development of immunosuppressive neutrophils and restrain the pro-inflammatory responses of neutrophils (73, 74). These inhibitory effects of androgens are also reported in ILC2. ILC2 in lung, GI tract and skin highly express AR compared to other lymphocytes and are negatively regulated by androgens. This is characterized by a low ILC2 cell number and low cytokine production in males compared with females (10, 11, 75, 76). Regarding conventional dendritic cells (cDCs), AR is barely expressed in these cell populations, but androgens may still affect DC activities indirectly. Studies have shown that androgen depletion enhances the expression of MHC-II and co-stimulatory molecules while reducing pro-inflammatory cytokine production (IL-1β, IL-6, and TNF-α) (77). Our recent study has demonstrated that androgens can reduce skin DC cell numbers by negatively regulating skin ILC2s, which could systemically downregulate skin immunity strength (78). The effects of androgen on different immune cells are summarized in **Figure 3**.

#### 3.1.2 Estrogens

The effects of female sex hormones on the immune system have also been extensively investigated, and a substantial body of evidence indicates that female sex hormones can enhance immune responses, which is correlated with the high incidence of autoimmune diseases in females. For example, E2 induces T-bet expression to favor Th1 differentiation and enhances IFNγ production (51, 79–81). E2 treatment also increases the activity of both CD8^+^ T cells and CD4^+^ T cells, along with cytokine production (79, 81–83). In the case of B cells, E2 treatment can elevate the level of autoreactive B cells and autoantibody production by reducing the potency for negative selection and promoting the expression of antiapoptotic genes such as *Bcl2*, thus favoring the survival of autoreactive B cells (84, 85). Estrogens potently induce the expression of activation-induced deaminase (AID), encoding by gene *Aicda*, to promote somatic hypermutation and class switch recombination (86). Estrogens also enhance the production of pro-inflammatory cytokines, such as type 1 IFN, IL-6, and TNF-α, in dendritic cells by amplifying TLR signaling (87, 88). E2 positively regulates neutrophil function in various aspects, including increasing neutrophil serine proteases and NO production, as well as enhancing the release of neutrophil extracellular traps (NET) (89–91).

However, many other studies have revealed that estrogen may also negatively regulate immune cell function and exhibit anti-inflammatory effects. For example, estrogens promote *Foxp3* expression to increase Treg levels and enhances the expression of PD-1 on Treg cells to protect against autoimmune diseases (92–95). Estrogens also promote STAT3 phosphorylation in macrophages, leading to an increased IL-10 production and favoring M2 polarization (96–98). In summary, estrogens have complex effects on the immune system, significantly influencing immune cell development, differentiation, and responses to stimuli (**Figure 3**).

#### 3.1.3 Progestogens

Progestogens, mainly progesterone (known as P4), are known for their anti-inflammatory effects on various types of immune cells (**Figure 3**). For instance, progesterone treatment inhibits the production of NO, IL-12, IL-1β, and TNF-α in macrophages while promoting M2 macrophage development (99–104). Similarly, progesterone limits DC activation, reducing proinflammatory cytokine production and inducing the production of anti-inflammatory cytokines (105–108). Moreover, progesterone differentially regulates the development of CD4^+^ T cell populations. A high level of progesterone increases the levels of Treg and Th2 cells and enhances their cytokine production, including IL-10, IL-4, and IL-5, but suppresses Th17 and Th1 cells (109–111). Progesterone also dampens the activation of NK cells and CD8^+^ T cells, reducing IFN-γ production [ (112–115). However, in some tissues, progestogens promote immune cell functions. For instance, progesterone treatment enhances decidual NK cell (uNK) migration as well as their effector function in the uterus (116, 117). progesterone also enhances neutrophil chemotaxis and transepithelial migration into the vaginal lumen, thereby promoting pathogen clearance (118, 119).

### 3.2 Intracellular mechanisms of sex hormone-regulated immune cells

**Figure 4:**
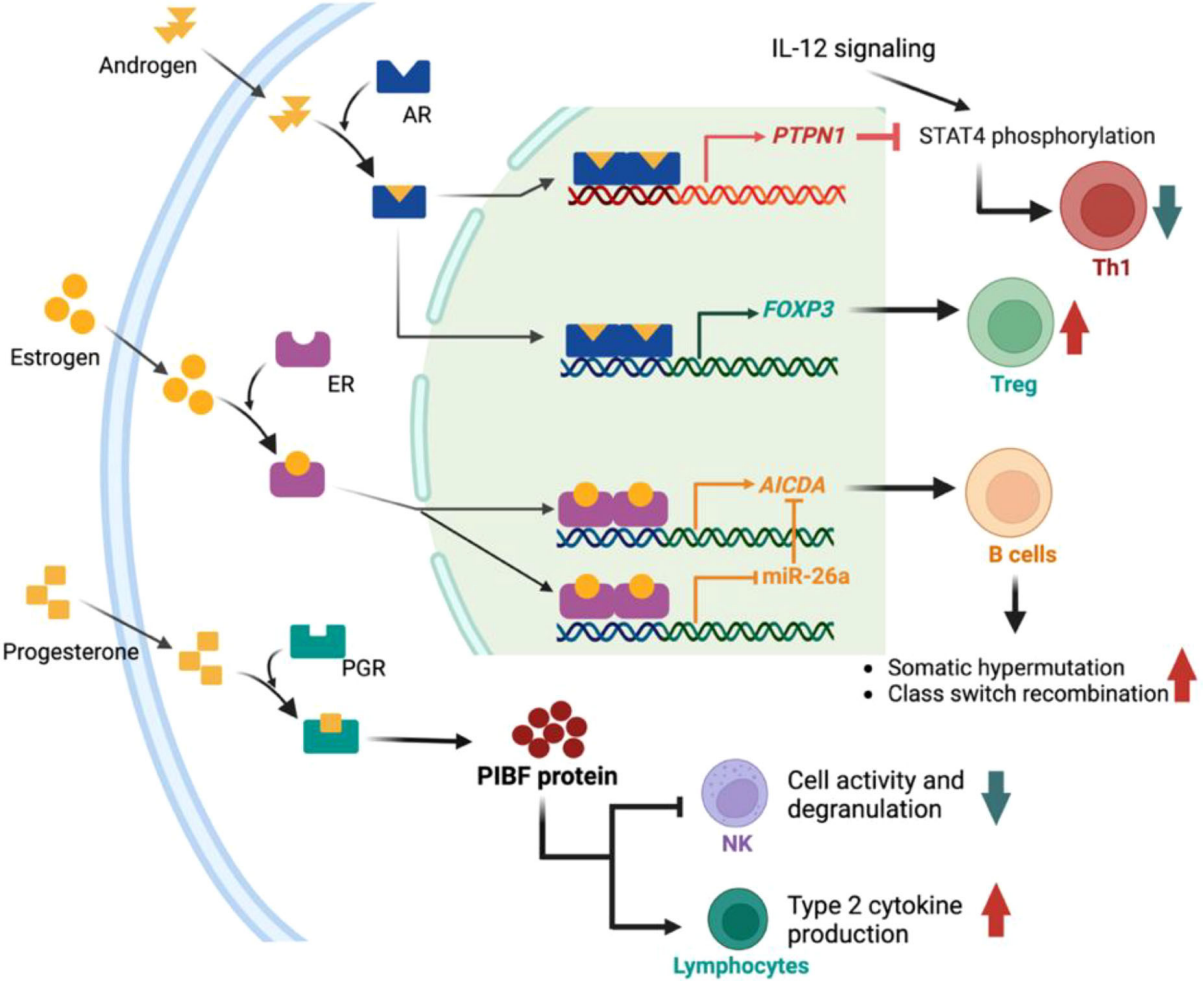
Several intracellular mechanisms of the sex hormone-immune crosstalk. AR signaling can suppress the Th1 development by promoting PTPN1 expression to inhibit IL-12 signaling, and AR signaling can promote Treg cells by enhancing *Foxp3* expression. Estrogens promote somatic hypermutation and class switch recombination of B cells by promoting *Aicda* expression. Progesterone induces Progesterone-induced blocking factor (PIBF) which increases type 2 cytokine production and decrease NK cell cytotoxicity.

Although the effects of sex hormones on various immune cells have been extensively reported, the intracellular mechanisms, particularly the direct transcriptional targets and epigenomic landscapes shaped by nuclear hormone receptors in immune cells, remain a significant knowledge gap. A major challenge lies in distinguishing cell-autonomous, receptor-mediated direct effects from indirect effects mediated by changes in the complex tissue microenvironment. The following sections discuss the limited examples where a direct regulatory mechanism has been convincingly established (**Figure 4**).

First, in CD4^+^ T cells, androgens activate AR which then binds to ARE region within gene *Ptpn1*, inducing increased expression of this gene. *Ptpn1* encodes a phosphatase that can dismiss the IL-12–induced STAT4 phosphorylation, thus suppressing Th1 differentiation (66). Likewise, in Treg cells, androgen-activated AR binds to the ARE within the intronic region of the *Foxp3* gene (64). The binding of AR changes the acetylation status of histone H4, induces the *Foxp3* gene expression, and causes the expansion of Foxp3^+^ Treg cells.

Additionally, estrogen-activated ERs can bind to the promoter of *Aicda* to enhance gene expression by over 20-fold (86). *Aicda* encoded AID is the key factor to initiate somatic hypermutation and class switch recombination in B cells. A recent study proposed another mechanism of estrogen regulated AID expression in the context of histone deacetylase inhibitor treatment (120). Histone deacetylase inhibitors increase the expression of miR-26a to silence AID expression in B cells. However, estrogen activated ERα can suppressed the miR-26a expression, thus reversing the histone deacetylase inhibitor-induced AID inhibition.

P4 is well known to promote lymphocytes to produce Progesterone-induced blocking factor (PIBF), a key downstream mediator with multiple immunomodulatory effects. The PIBF receptor is associated with the alpha chain of the IL-4 receptor, in which PIBF signaling promotes STAT6 activation and then boost the Th2 cytokine production, such as IL-3, IL-4, and IL-10, to regulate Th1/Th2 balance (121, 122). Moreover, PIBF signaling can inhibit the activity and degranulation of NK cells by suppressing perforin expression (123, 124). PIBF-mediated immunomodulatory effects play critical role in maintaining immune tolerance during pregnancy, and dysfunction of PIBF signaling is associated with pregnancy failure (125, 126).

These examples above underscore that sex hormones can orchestrate immune cell function by directly rewiring transcriptional and epigenetic programs. However, our current map of these direct targets is remarkably sparse. To achieve a mechanistic understanding, future work needs to define the direct targets and epigenetic landscape of sex hormones in immune cells. Employing cell type-specific genomic techniques, such as hormone receptor ChIP-seq coupled with ATAC-seq or DNA methylation analysis, will be key to get a systems-level view of their immunomodulatory actions.

### 3.3 Determinants of context-dependent and conflicting effects of sex hormones on immune cells

While the overall interaction between sex hormones and the immune system is well-established, inconsistencies in the effects of sex hormones on the immune system among different studies may be influenced by several factors.

#### 3.3.1 Dose-dependent effects

The dosage of sex hormones may profoundly impact their physiological effects, and many studies have found that the impact of sex hormones on the immune system is dose dependent. For example, low levels of E2 enhance Th1 differentiation and IFNγ production, while high levels of E2 decrease IFN-γ production but increase IL-4 production to favor Th2 differentiation (79, 81, 127). An *in vitro* studies have shown that lower concentrations of E2 enhance T lymphocyte proliferation, IFN-γ, and NO production, while higher doses suppress these effects (128). The dose-dependent effects of estrogens on immune cells may also be related to the changes in NK cell activity during pregnancy. NK cell activity significantly decreases during the second/third trimester of pregnancy when estrogen levels are greatly elevated compared to the first trimester or non-pregnant states (129, 130). Progesterone may also exhibit dose-dependent effects; one *in vitro* study found that placenta-relevant levels (high dose) of progesterone inhibited T cell activation, while serum-relevant progesterone levels enhanced T cell activation and protein secretion (83). Likewise, low levels of progesterone (0.2-2 μg/mL) increase IFN-α production in pDCs, but high progesterone (20 μg/mL) significantly inhibits it (131). Similarly, some immune effects of androgens are also dose de pendent. For example, DHT only suppresses the cytotoxicity of NK cells against castration-resistant prostate cancer cells at a high dose level (50 nM), while doses of 1 nM and 10 nM do not show significant effects. These studies suggest that the immune effects of sex hormones are highly dose-dependent, emphasizing the need to consider and specify the dosage levels in future studies.

#### 3.3.2 Receptor selection

Another important factor contributing to the diverse effects of sex hormones is receptor selection. The same hormone molecules may induce different effects in immune cells by interacting with different types or subtypes of receptors. As discussed in Section 1.3, ERα and ERβ have distinct tissue expression preferences and some biological effects. These receptor-specific-effects of estrogen are also observed in immune cells. For instance, ERα and ERβ have been suggested to have opposing effects on immune cell proliferation and apoptosis. ERα signaling stimulates proliferation and has anti-apoptotic effects, while ERβ signaling suppresses proliferation and induces apoptosis (132). ERα and ERβ also play distinct roles in regulating B-cell maturation and selection. Both ERα and ERβ signaling can regulate the E2-induced changes in transitional B-cell numbers, but only the activation of ERα signaling de creases BCR signaling pathways (133). Different effects of ERα and ERβ signaling have also been observed in the context of collagen-induced arthritis, an autoimmune model (134). As described, progesterone can bind with both PR and GR, each of which has distinct effects on immune cells. For instance, progesterone reduces NO production in macrophages by activating GR signaling but not PR signaling (103). Similarly, progesterone exclusively inhibits TLR3- and TLR4-induced IL-6 production by activating GR signaling (100). Therefore, some of the immune effects of sex hormones are largely determined by the receptors they activated and the subsequent intracellular pathways they engaged.

Many other factors may also influence sex hormone effects on immune cells, such as tissue microenvironments, disease contexts, or even the choice of animal modes. However, our current knowledge in this area is still limited and requires further exploration in future studies.

## 4 Epithelial and stromal cells: A bridge between sex hormones and immune cells

The epithelial/stromal–immune cell interactions play critical roles in various biological processes, such as immune responses to pathogen infections, anti-tumor immunity, and tissue maintenance and repair (135). Besides providing structural support and nutrients, epithelial/stromal cells release signals that coordinate the activities of the local immune system, such as supporting the survival and activation of tissue-resident or infiltrating immune cells. For example, they produce chemokines that promote immune cell infiltration and guide T and B cells to secondary lymphoid organs (136, 137). In the thymus, mTECs and cortical thymic epithelial cells (cTECs) release cytokines and tissue-restricted antigens to facilitate the proliferation of developing T cells and coordinate their positive and negative selection processes (138). Furthermore, recent research has shown that mesenchymal stromal cells can instruct neighboring macrophages to increase their phagocytic activity and amphiregulin production through the release of PGE2, which, in turn, promotes tissue remodeling and maturation (139).

### 4.1 Sex hormones regulate the immune system by signaling through epithelial and stromal cells

Sex hormone receptors are expressed on epithelial/stromal cells in various tissues, such as thymus (140, 141), skin (142), and lung (143, 144). Given the essential role of epithelial/stromal cells in regulating immune cells, the crosstalk between sex hormones and epithelial/stromal cells may be an important mechanism through which sex hormones indirectly regulate the immune system. Several studies have found sex hormones can regulate the immune system by targeting epithelial/stromal cells. For example, mice engrafted with bone marrow cells expressing the androgen receptor (AR) exclusively in thymocytes showed thymic enlargement and androgen insensitivity, while mice engrafted with bone marrow cells expressing AR exclusively in epithelial cells but not thymocytes exhibited normal thymus size and a typical involutional response to androgens (141). This suggests that androgen-induced reduction of lymphoid cells in the thymus depends on androgen signaling in thymic epithelial cells. Additionally, as described, AR signaling also promotes the expression of *Aire* in medullary thymic epithelial cells, facilitating self-antigen expression and the induction of negative selection in T cells (62). A similar phenomenon is observed in bone marrow, where androgens suppress B cell development by activating AR signaling in stromal cells, leading to the production of TGF-β (145). An *in vitro* study found that estrogen regulated B lymphopoiesis only in the presence of stromal cells though estrogen receptors are expressed in both B cells and stromal cells (146). Therefore, epithelial/stromal cells can serve as essential mediators that facilitate communication between sex hormones and immune cells.

### 4.2 Epithelial/stromal cells synthesize sex hormones to regulate tissue-resident immune cells

**Figure 5:**
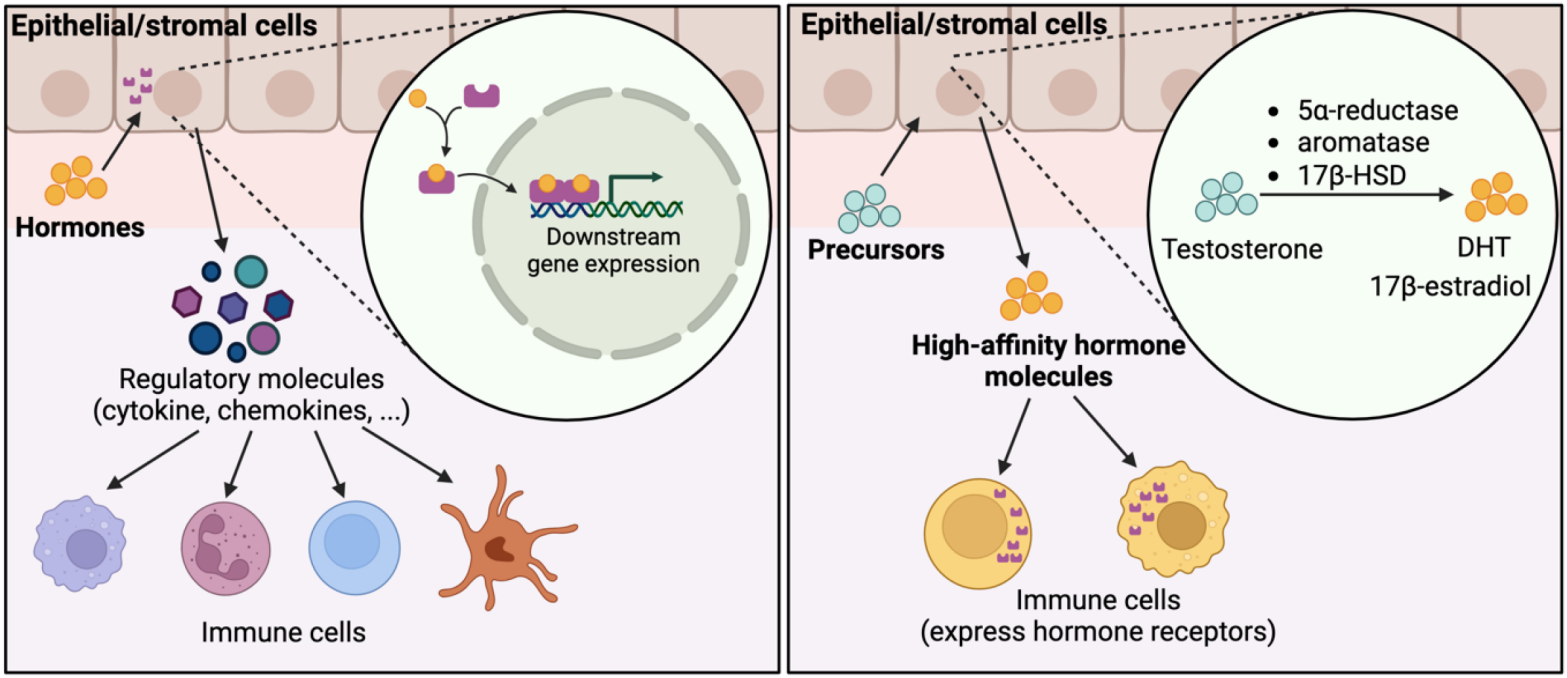
A framework for understanding the crosstalk among sex hormones, epithelial/stromal cells, and immune cells. Sex hormones can activate sex hormone receptors expressed in stromal cells which then regulate the production of functional molecules, such as cytokines and chemokines, which could further influence the activity of immune cells. On the other hand, stromal cells express enzymes, such as 5α-reductase, aromatase, and 17β-HSD, which can utilize hormone precursors to synthesize high-affinity hormone molecules. These hormone molecules then regulate the immune cells expressing hormone receptors.

As previously discussed, peripheral tissues can synthesize high levels of sex hormones, significantly influencing the local tissue microenvironment. Epithelial and stromal cells play a dominant role in this process, as they express various hormone synthetic enzymes. For instance, skin epithelial cells, such as keratinocytes, express several steroidogenic enzymes involved in converting DHEA/DHEAS (DHEA sulfate) into testosterone, estradiol, and DHT. These enzymes include 17β-hydroxysteroid dehydrogenase (17β-HSD), steroid 5α-reductases, and aromatase (**Figure 5**) (147). Another example is the syncytiotrophoblast in the placenta, which produces substantial quantities of estrogens and progesterone, crucial for maintaining pregnancy and fetal growth (148).

Tissue synthesized sex hormones may play a pivotal role in shaping tissue-specific immune responses and contribute to the varying degrees of sex disparity seen among different tissues. Sex hormones synthesized in peripheral tissues primarily act locally, creating tissue-specific hormone concentrations and unique microenvironments (149). This can elevate sex hormone levels locally while systemic hormone levels remain low, enhancing the effects of sex hormones on immune cells resident in these tissues. For example, while testosterone and DHT levels are typically low in females’ bodies systemically, high expression of 5-reductases in skin epithelial cells can lead to significant local synthesis of DHT from precursors, potentially exerting important effects on the immune system in the skin. Variations in sex hormone synthesis among tissues may be an important factor contributing to the sex-specific immune responses observed in different tissues. A recent study has revealed that sex-related differences in gene expression across human tissues exhibit considerable variability, with immune response-related pathways displaying distinct sex differences depending on the tissue involved (150).

Despite these insights, our current understanding of the effects of tissue-specific sex hormone synthesis on the local immune system remains limited and necessitates further investigation in future studies.

## 5 Factors modulating sex hormone effects on the immune system

We next discuss several factors that can significantly impact the crosstalk between sex hormones and the immune system. We emphasize the importance of understanding how fluctuations in sex hormone levels, driven by both internal and external factors, can influence interactions with the immune system, subsequently affecting body homeostasis. Four key regulatory factors are focused: pregnancy, aging, the microbiota, and exposure to hormone disruptors. These factors can directly influence sex hormone levels but their impacts on the sex hormone-immune system crosstalk haven’t been well understood.

### 5.1 Pregnancy

As displaying in **Figures 1E**, pregnancy is associated with dramatical sex hormone changes, including estrogens and progesterone, which are essential for a successful pregnancy. Simultaneously, immune system is known to experience profoundly remodeling during pregnancy. As described, both estrogens and progesterone can impact the immune system, thus the changes of sex hormone level plays an key role in these pregnancy-associated immune remodeling (151). For instance, Treg cells are expanded during pregnancy which is critical to maintain feto-maternal tolerance and promote fetal survival (152–154). E2 treatment mimic the pregnancy-associated enhancement of Foxp3 expression and Treg cell function (155, 156). Furthermore, pregnancy is marked by the expansion of T-helper 2 (Th2) cells and the upregulation of anti-inflammatory cytokines such as IL-4 and IL-10, while pro-inflammatory cytokines like TNFα and IFN-γ decrease (157, 158). The elevated PIBF, which is produced by lymphocytes with the stimulation of progesterone, may play an important role in the enhanced Th2 cells and the associated cytokine profiles during pregnancy (159, 160). Uterine natural killer cells (uNKs), the most abundant lymphocytes in the uterus, are vital for the establishment of a successful pregnancy. Importantly, ERs are expressed in uNKs and estrogens facilitate uNK migration and uNK-mediated angiogenesis by augmenting the secretion of CCL2 (161). Notably, the immune remodeling during pregnancy has far-reaching consequences, altering the susceptibility and severity of various diseases. Pregnant individuals may experience reduced severity of inflammatory diseases while becoming more susceptible to infections. Comprehensive insights into these changes in the immune system and their effects on disease susceptibility are provided in previous reviews (151, 162–164).

However, in addition to sex hormones, the body is marked by the changes of many other factors, making it challenging to isolate the distinct role of sex hormones in pregnancy-related immune remodeling. Therefore, the specifical roles and mechanisms of sex hormone-induced immune changes during pregnancy are still not fully understood and warrant in-depth investigation. Moreover, late pregnancy relies heavily on the placenta as the primary source of sex hormones and subsequent to childbirth, with the expulsion of the placenta, sex hormone levels undergo a rapid and dramatic decline. The impacts of such abrupt hormonal shifts on the maternal immune system and their potential impact on disease pathogenesis have received limited scrutiny to date. It also needs to be further investigated that how the immune remodeling regresses after pregnancy and lactation and how this process impact maternal health.

Another intriguing question is that whether fetal sex could influence the maternal immune system. A study has found that fetal sex is associated the LPS-stimulated cytokine production of PBMCs from pregnant women, that women with female fetuses exhibited greater stimulated cytokine production (165). Another study also found some association between female fetal sex and cytokine level in maternal blood (166). An recent study observed that the quality of antibody responses to SARS-CoV-2 infection and immunization are also different between pregnant women with female and male fetuses (167). However, the precise impact of fetal sex on the maternal immune system and the potential involvement of fetal sex hormones in this process remain largely obscure. Future studies are needed to provide a more comprehensive understanding of the intricate relationship between sex hormones, maternal immunity, and fetal sex in the context of pregnancy.

### 5.2 Aging

Aging is associated with the misfunction of the immune system, usually characterized as the increase of chronic low-grade inflammation, reduction of lymphopoiesis and the functional declines of multiple immune cells, such as Th17 cells, B cells, and neutrophils. These age-related immune changes can deeply impact susceptibility to diseases, particularly autoimmune diseases and cancers, as well as affect the efficacy of vaccines. As described earlier, sex hormone levels decline significantly during the aging process in both males and females, which can profoundly influence the homeostatic crosstalk between hormones and the immune system. Some studies have identified some immune changes that relevant to the decline in sex hormones during aging. For example, hormone replacement therapy with estrogen and progesterone in late postmenopausal women has been shown to mitigate the age-related decline in peripheral B-2 cells (168). Hormone replacement therapy can also shift the cytokine balance in aging women by decreasing the levels of certain cytokines, including IFN-γ, TNFα, IL-10, IL-2, and IL-4 (169–171). Similarly, androgen supplementation has demonstrated the potential to ameliorate age-induced changes in the frequency of naïve and memory T cells and inflammatory cytokine levels in male rhesus macaques (172). Testosterone treatment has partially improved infection-induced morbidity and mortality and restored pulmonary cytokine responses in aging males (173). Sex hormones play an important role in shaping sex differences in immune responses and disease outcomes, but intriguingly, the decline of sex hormones during aging is not necessarily reduce sex differences in the immune system. A recent study has even suggested that sex biases in human peripheral blood mononuclear cells (PBMCs) increase after the age of 65 (174), indicating the influence of other factors in shaping sex differences in the aging immune system. This finding may also suggest a dose-dependent effect of sex hormones.

In summary, the interplay between sex hormones and the immune system undergoes significant changes in the context of aging. Studying this interaction in the context of aging will enhance our understanding of the mechanisms behind age-related immune changes and associated diseases. This knowledge may lead to the development of new interventions or therapeutic approaches.

### 5.3 Pathological disruption of sex hormone homeostasis

In addition to physiological fluctuations of sex hormones, some pathological conditions with dysregulation of the HPG axis and sex hormone homeostasis also need to be concerned which provide critical *in vivo* models of chronic hormone–immune imbalance. For example, polycystic ovary syndrome (PCOS), characterized by hyperandrogenism, are associated with systemic low-grade inflammation, altered monocyte/macrophage activity, a skewed lymphocyte balance, and changed level of cytokines (175–177). Likewise, endometriosis, an estrogen-driven disorder, features an elevation of proinflammatory cytokines, impaired NK cell function, and heightened infiltration of proinflammatory macrophages (178–181). These clinical syndromes exemplify how sustained endocrine imbalance can profoundly affect immune landscape, directly contributing to disease pathogenesis and complications. Studying these states not only elucidates disease mechanisms but also offers unique insights into the long-term consequences of a dysregulated sex hormone-immune axis.

### 5.4 Sex hormone therapy

Exogenous hormone administration, such as menopausal hormone therapy, gender-affirming hormone therapy and androgen deprivation therapy (ADT), induces distinct immune changes that reflect the biological activity of the administered hormones. Menopausal hormone therapy has been associated with immune changes such as modulated cytokine profiles and altered B cell and NK cell populations (182, 183). Testosterone therapy in transgender men has been shown to attenuate type-I interferon responses while enhancing monocyte inflammatory activity (184). Conversely, androgen deprivation therapy for prostate cancer leads to an expansion of the naive T-cell compartment, increase in activated CD8 T cells and proinflammatory M1-like tumor-associated macrophages (185–187). These findings illustrate that therapeutic manipulation of sex hormone levels can profoundly redirect immune responses. Further longitudinal studies to define the precise immunological trajectories induced by such interventions will be essential for advancing both our mechanistic understanding of hormone-immune crosstalk and optimizing therapy strategies.

### 5.5 Microbiota

Microbiota is deeply involved in various host physiological processes from metabolic homeostasis and immune balance. Recent studies have revealed that microbiota can also directly regulate the level of sex hormones in the host. First, similar with bile acid metabolism, gut bacteria with β-glucuronidase can deconjugate the β-D-glucuronic acid-conjugated hormone molecules (estradiol, testosterone, and DHT), releasing the active forms of these hormones in the gut (188, 189). This microbiota-mediated reactivation of sex hormones can lead to exceptionally high hormone levels locally. For instance, young adult men may have a significantly higher level of unconjugated DHT in the gut—up to 70 times higher than in their sera (189). This local increase in hormone levels could profoundly affect epithelial cells, stromal cells, and immune cells expressing AR. Conversely, a recent study has also found *Mycobacterium neoaurum*, a bacterium isolated from fecal samples of testosterone-deficient patients, express 3β-hydroxysteroid dehydrogenase which has distinct capability to degrade testosterone (190). Treating animals with 3β-HSD-producing bacteria has been shown to lower testosterone levels and induce depression-like behaviors. Intriguingly, the degradation of testosterone in the gut significantly reduces testosterone levels both in the serum and in the brain. This suggests that microbiota-mediated sex hormone metabolism can have systemic effects, potentially impacting remote immune systems. Moreover, the microbiota can produce sex hormone antagonists that negatively regulate hormonal signaling. For instance, a recently identified microbiota-derived bile acid metabolite exhibits potent AR antagonistic activity, thereby ablating androgen-mediated effects on CD8^+^ T cells (69). Taken together, gut microbiota can produce various enzymes that either increase or decrease sex hormone levels. However, our current understanding of the impact of microbiota on sex hormone levels is still limited. Additionally, how microbiota-mediated sex hormone metabolism influences the homeostasis of local and remote immune systems remains largely unclear. Further research is needed to address these questions and gain a more comprehensive understanding of the complex interplay between microbiota, sex hormones, and the immune system.

### 5.6 Sex hormone disruptors

Given the pivotal role of sex hormones in immune system regulation, it’s important to consider the impact of exposure to chemicals that disrupt sex hormone homeostasis on the immune system. These chemicals, known as endocrine disruptors, can influence sex hormone signals through various mechanisms, including directly binding to sex hormone receptors, thereby enhancing or disrupting hormone signaling. Some chemicals found in food, like genistein and daidzein (two isoflavones in soybeans known as phytoestrogens), have structures similar to hormone molecules (191). Genistein, for instance, exhibits binding affinity to multiple estrogen receptors, including ERα, ERβ, and GPER, while daidzein can bind to GPER and influence ERβ expression levels. Industrial chemicals, such as Bisphenol A (BPA), phthalates, octylphenol, and Dichlorodiphenyltrichloroethane (DDT), are also endocrine disruptors that can bind to sex hormone receptors. **Table 1** provides examples of some common endocrine disruptors and their target sex hormone receptors.

**Table 1:** Typical endocrine-disrupting chemicals and their targeted sex hormone receptors.

Chemicals	Source	Targeted hormone receptors	References
Bisphenol A	Polycarbonates, plastics, and so on.	AR, ERα and ERβ	(207–210)
Bisphenol AF	Cosmetics, plastics and so on.	AR, ERα and ERβ	(207, 209–211)
Genistein	Plants	ERβ	(212)
Daidzein	Plants	ERβ	(212)
4-tert-Octylphenol	Detergents, cleaners, and emulsifiers	AR, ERα and ERβ	(210)
4-Nonylphenol	Antioxidants, lubricating oil, detergents, emulsifiers, and solubilizers	ERα and ERβ	(213–215)
DTT	Insecticide	ERα	(216)
Ditridecyl phthalate	Building materials, personal care products, detergents pharmaceuticals, food products	ERα and ERβ	(217)
Benzylbutyl phthalate	Vinyl foams	AR, ERα and ERβ	(217)

Exposure to these chemicals may disturb immune homeostasis and lead to diseases. Numerous studies have shown that sex hormone disruptors can affect various aspects of the immune system, including immune cell development, proliferation, and cytokine production (192–194). For example, BPA and its derivatives, like Bisphenol AF, are widely studied endocrine disruptors used in plastics manufacturing. These compounds bind to ERα, ERβ, and GPER, albeit with lower affinity compared to 17β-estradiol (195, 196). BPA has been shown to share some immune effects with estrogens, such as enhancing autoantibody production in B1 cells (197) and inducing hyperprolactinemia in a genetically predisposed rat model by increasing PRL regulating factor activity in the posterior pituitary (198). Nonylphenol, which exhibits estrogen-like activity, has effects similar to estradiol in increasing TNF-α expression in human dendritic cells (199). Octylphenol, which is used to manufacture anionic surfactants, also has a weak binding affinity to ERs but have antiestrogenic activity potentially by competitive binding (200). Exposure to octylphenol can decrease estradiol-enhanced IL-1β expression in a monocytic cell line in the context of LPS stimulation (201), on the other hand, estradiol could protect against octylphenol-induced apoptosis in cultured splenocyte (202). In addition, BPA, octylphenol and nonylphenol can suppress LPS-induced NO production NF-κB activation in macrophages through a ER-dependent mechanism (203).

However, it’s crucial to note that endocrine-disrupting chemicals can have complex toxic effects. Immune changes induced by these chemicals may not solely result from disrupting sex hormone signaling. For example, genistein can induce the activation of eNOS/NO axis which is independent with the ER signaling (204). Endocrine disruptors also can interact with other transcription factors and regulators beyond sex hormone receptors, such as AhR, ERRγ, and PPARγ (205, 206). In summary, exposure to endocrine-disrupting chemicals can have significant effects on immune system function by perturbing sex hormone signaling. However, the mechanisms underlying these effects can be complex and multifaceted, necessitating additional research to elucidate the precise interactions among these factors.

## 6 Conclusion

In conclusion, the intricate interplay between sex hormones and the immune system represents a multifaceted regulatory network that significantly impacts various aspects of health and disease. This dynamic relationship extends from immune cell development and function to tissue-specific immune responses, affecting susceptibility to autoimmune disorders, infections, and cancers. While substantial progress has been made in deciphering the roles of sex hormones in immunity, many questions remain unanswered. Future research endeavors should aim to elucidate the intricate mechanisms underlying sex hormone-mediated immune modulation, including the molecular pathways activated within immune cells in response to sex hormone signaling. The critical roles of epithelial and stromal cells in the sex hormone-immune crosstalk need to be specially concerned. Moreover, the influence of factors such as microbiota, pregnancy, aging, and exposure to endocrine-disrupting chemicals on the sex hormone-immune crosstalk warrants further investigation. A comprehensive understanding of these interactions is pivotal for the development of tailored therapeutic strategies and the realization of personalized medicine, ultimately leading to improved healthcare outcomes for individuals of all sexes.
